# Readmission Rates After Acute Respiratory Distress Syndrome in Children

**DOI:** 10.1001/jamanetworkopen.2023.30774

**Published:** 2023-09-08

**Authors:** Garrett Keim, Jesse Y. Hsu, Neethi P. Pinto, Megan L. McSherry, Annie Laurie Gula, Jason D. Christie, Nadir Yehya

**Affiliations:** 1 Department of Anesthesiology and Critical Care Medicine, Children’s Hospital of Philadelphia, Philadelphia, Pennsylvania; 2 Department of Anesthesiology and Critical Care, Perelman School of Medicine, University of Pennsylvania, Philadelphia; 3 Leonard Davis Institute of Health Economics, University of Pennsylvania, Philadelphia; 4 Center for Clinical Epidemiology and Biostatistics, Perelman School of Medicine, University of Pennsylvania, Philadelphia; 5 Division of Pulmonary, Allergy and Critical Care Medicine, Hospital of the University of Pennsylvania, University of Pennsylvania Perelman School of Medicine, Philadelphia

## Abstract

**Importance:**

An increasing number of children survive after acute respiratory distress syndrome (ARDS). The long-term morbidity affecting these survivors, including the burden of hospital readmission and key factors associated with readmission, is unknown.

**Objective:**

To determine 1-year readmission rates among survivors of pediatric ARDS and to investigate the associations of 3 key index hospitalization factors (presence or development of a complex chronic condition, receipt of a tracheostomy, and hospital length of stay [LOS]) with readmission.

**Design, Setting, and Participants:**

This retrospective cohort study used data from the commercial or Medicaid IBM MarketScan databases between 2013 and 2017, with follow-up data through 2018. Participants included hospitalized children (aged ≥28 days to <18 years) who received mechanical ventilation and had algorithm-identified ARDS. Data analysis was completed from March 2022 to March 2023.

**Exposures:**

Complex chronic conditions (none, nonrespiratory, and respiratory), receipt of tracheostomy, and index hospital LOS.

**Main Outcomes and Measures:**

The primary outcome was 1-year, all-cause hospital readmission. Univariable and multivariable Cox proportional hazard models were created to test the association of key hospitalization factors with readmission.

**Results:**

One-year readmission occurred in 3748 of 13 505 children (median [IQR] age, 4 [0-14] years; 7869 boys [58.3%]) with mechanically ventilated ARDS who survived to hospital discharge. In survival analysis, the probability of 1-year readmission was 30.0% (95% CI, 29.0%-30.8%). One-half of readmissions occurred within 61 days of discharge (95% CI, 56-67 days). Both respiratory (adjusted hazard ratio [aHR], 2.69; 95% CI, 2.42-2.98) and nonrespiratory (aHR, 1.86; 95% CI, 1.71-2.03) complex chronic conditions were associated with 1-year readmission. Placement of a new tracheostomy (aHR, 1.98; 95% CI, 1.69-2.33) and LOS 14 days or longer (aHR, 1.87; 95% CI, 1.62-2.16) were associated with readmission. After exclusion of children with chronic conditions, LOS 14 days or longer continued to be associated with readmission (aHR, 1.92; 95% CI, 1.49-2.47).

**Conclusions and Relevance:**

In this retrospective cohort study of children with ARDS who survived to discharge, important factors associated with readmission included the presence or development of chronic medical conditions during the index admission, tracheostomy placement during index admission, and index hospitalization of 14 days or longer. Future studies should evaluate whether postdischarge interventions (eg, telephonic contact, follow-up clinics, and home health care) may help reduce the readmission burden.

## Key Points

**Question:**

What factors are associated with readmission in pediatric survivors of acute respiratory distress syndrome (ARDS)?

**Findings:**

In this cohort study of 13 505 children who survived ARDS, the probability of hospital readmission within 1 year was 30.0%, with one-half of readmissions occurring within 61 days of discharge. The presence of a complex chronic condition, hospitalization for ARDS lasting 14 days or longer, and receipt of a tracheostomy during the index hospitalization were all associated with increased rates of 30-day, 60-day, and 1-year readmission.

**Meaning:**

These findings suggest that childhood survivors of ARDS are at high risk of readmission in the first 2 months after discharge; future studies should evaluate whether postdischarge interventions (eg, telephonic contact, follow-up clinics, and home health care) may help reduce the readmission burden.

## Teaser

This cohort study examines 1-year readmission rates among survivors of pediatric acute respiratory distress syndrome and investigates the associations of 3 key index hospitalization factors (presence or development of a complex chronic condition, receipt of a tracheostomy, and length of stay) with readmission.

## Introduction

More than 100 000 children annually in the US require mechanical ventilation (MV) for the management of acute respiratory failure.^1,2,3^ Despite increasing survivorship among children with acute respiratory distress syndrome (ARDS) and extensive publications^4,5^ highlighting lasting health and quality of life detriments in adult ARDS survivors, the long-term impact on children is less known.

One important outcome of interest is hospital readmission. Hospital readmission affects children, their families, and the health care system.^6^ Readmission increases overall hospital costs and hospital days for affected children.^7^ Increased hospital days leading to school absenteeism has been associated with worse educational outcomes for children with asthma.^8^ Unplanned readmissions decrease the number of available inpatient beds in a system where bed shortages are becoming more frequent.^9^ For patients requiring pediatric intensive care unit (PICU) admission, 11% were readmitted to the same PICU in the following year.^10^ In a separate study,^11^ nearly one-half of pediatric survivors of severe sepsis were readmitted within 1 year. Previous studies^12^ of children found that new or increased medical complexity at the index hospital discharge was associated with increased odds of readmission. Determination of readmission after MV and pediatric respiratory failure has been limited to evaluating readmissions to the same hospital,^13,14^ which does not account for patients readmitted to different hospital systems after the index admission,^15^ and has limited generalizability. Therefore, by using insurance-based administrative data sets to overcome limitations of previous studies and leveraging a validated algorithm based on *International Classification of Diseases, Ninth Revision (ICD-9) *and *International Statistical Classification of Diseases and Related Health Problems, Tenth Revision (ICD-10)* codes to identify pediatric ARDS,^16^ we aimed to determine the burden of 1-year readmission in survivors of ARDS. We assessed 3 key index hospitalization exposures—(1) the presence of a complex chronic condition (CCC)^17,18^ at discharge, (2) receipt of a new tracheostomy, and (3) index hospitalization length of stay (LOS)^18^—for their association with 1-year readmission in survivors of algorithm-identified ARDS. We hypothesized that readmission would occur in one-quarter of survivors and that the presence of a respiratory CCC, receipt of a tracheostomy during index admission, and longer LOS would be associated with increased hospital readmission after discharge.

## Methods

### Cohort Development

We performed a retrospective cohort study of children (aged ≥28 days to <18 years) with a respiratory illness or the presence of MV or endotracheal intubation (eTable 1 in Supplement 1) in the commercial or Medicaid IBM MarketScan databases between 2013 and 2018. MarketScan is an administrative insurance claims database representing 49 states with comprehensive records for inpatient, emergency department, and outpatient data for more than 43 million claimants.^19^ This study was deemed exempt, and Health Insurance Portability and Accountability Act authorization was waived by the Children’s Hospital of Philadelphia institutional review board. The board also waived the need for informed consent because the data were anonymous and not considered human participants research, in accordance with 45 CFR §46. We used the Strengthening the Reporting of Observational Studies in Epidemiology (STROBE) reporting guideline when writing our report.^20^ We excluded children without 30 days of insurance coverage before the index hospitalization admission date to exclude potential midhospitalization insurance enrollments that could have affected accuracy of LOS, one of our primary exposures of interest. We considered index admissions from 2013 to 2017 to allow for 1-year follow-up data through 2018. There was no missingness among variables included in proportional hazard analyses.

We determined both the presence and categorization of CCCs using Feudtner et al^17^ and the CCC version 2 statistical package^21^ from available prehospital *ICD-9* and *ICD-10* codes in the 30 days before admission, if available (207 admissions), and/or from index hospitalization discharge *ICD-9* and *ICD-10* codes. Using categorization developed by Feudtner et al,^17^ we delineated a 3-level categorical CCC exposure of none, respiratory CCC, and nonrespiratory CCC for use as one of the primary exposures in hazard modeling. We used both *ICD-9* and *ICD-10* codes because the study periods spanned the coding conversion in 2015. We determined the presence of algorithm-identified ARDS as children with acute respiratory failure requiring MV from a pulmonary parenchymal, sepsis, or shock source requiring MV for 24 hours or longer using our previously published validated *ICD-10* coding algorithm^16^ (eTable 1 in Supplement 1).

### Outcomes

Our primary outcome was hospital readmission within 1 year after index hospitalization, determined by a new hospitalization record on or before 365 days after the index discharge date. Readmissions on the same day as the index discharge were excluded. Patients with a discharge disposition of *transfer to another acute care facility* or *still patient* were queried for separate hospitalization record with the same date as the day of discharge, and these 2 admissions were combined as index hospitalization; if no new admission record could be found on the same date of discharge, these patients were excluded (1 patient). We determined readmission probabilities at each time point of interest using Kaplan-Meier survival analysis. We censored patients at 366 days or at the time of insurance coverage loss if occurring before 365 days. We performed sensitivity analyses using secondary outcomes of hospital readmission at 30 and 60 days. We assessed days alive and out of hospital (DAOH) after discharge as the cumulative days alive and not admitted to a hospital in the first 365 days after index hospitalization discharge.

### Power

We determined this study was adequately powered with an available cohort of 13 505 participants. We required a minimum sample size of 3853 participants to detect a clinically meaningful 5% difference in readmission between nonrespiratory and respiratory CCC, with a 3:1 ratio of patients with nonrespiratory to respiratory CCC and a power of 0.8 at significance level of *P* < .05.

### Statistical Analysis

We conducted analyses using Stata statistical software version 17 (StataCorp, LLC). We presented continuous data as medians and IQRs and binary and categorical data as frequency percentages. We analyzed continuous data using Wilcoxon rank-sum or Kruskal-Wallis tests and categorical data with Pearson χ^2^ test; all tests were 2 sided. Significance was set at *P* < .05. We plotted unadjusted Kaplan-Meier survival curves with the failure function representing the proportion of patients readmitted by a certain time point. We performed both univariable and multivariable Cox proportional hazard modeling. Assumptions of proportional hazards were tested by plotting log cumulative hazard function and comparing estimated vs true hazard curves. In addition, we performed sensitivity analyses using an exponential distribution and with the outcome of readmission at 30 and 60 days to address concerns of the potential time-varying nature of readmission at 1 year. Asthma diagnosis was a component of the algorithmic ARDS definition, yet asthma readmission risk factors have been extensively studied.^22^ We performed a subanalysis excluding 288 children with asthma to examine whether ARDS readmission associations were altered by the presence of asthma. We constructed a directed acyclic graph to select important confounders (age, immunocompromised status as defined by *ICD-10* codes for immunocompromising condition, need for rehabilitation as defined by discharge disposition, and lower socioeconomic status using Medicaid insurance as a surrogate) (eFigure 1 in Supplement 1). We tested the 3 key index hospitalization exposures of interest (the presence of CCC, LOS, and new tracheostomy) on the basis of prior risk factors identified in previously published studies^23,24^ and candidate factors that may represent ARDS course severity in separate multivariable Cox models because of the concern for collinearity between CCC and LOS. We assessed DAOH after discharge using linear regression models. We assumed survival until readmission or censoring for all patients in DAOH calculations because outpatient mortality data were unavailable. Data analysis was completed from March 2022 to March 2023.

## Results

**Figure 1.**
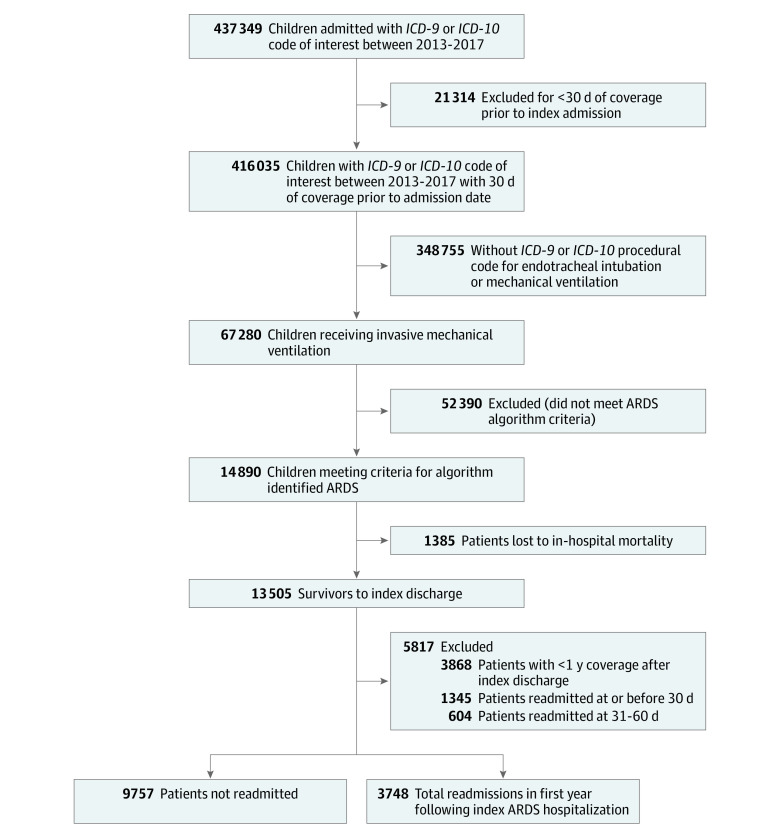
Participant Enrollment Flowchart ARDS indicates acute respiratory distress syndrome; *ICD-9*, *International Classification of Diseases, Ninth Revision*; *ICD-10*, *International Statistical Classification of Diseases and Related Health Problems, Tenth Revision*.

A total of 14 890 children were identified as having ARDS with an in-hospital mortality of 9.3% (1385 children) (Figure 1). Of the 13 505 children who survived to hospital discharge (median [IQR] age, 4 [0-14] years; 7869 boys [58.3%]), readmission within 1 year after ARDS occurred in 27.8% (3748 children) (Table 1). LOSs differed for readmitted children compared with those who were not readmitted (median [IQR], 19 [7-55] days vs 10 [4-28] days) (eFigure 2 in Supplement 1). The presence of 1 or more CCC, identified from either prehospital or discharge records, was significantly higher among children readmitted compared with those not readmitted (2895 of 3748 children [77.3%] vs 5654 of 9757 children [63.3%]; Pearson χ^2^_1_ = 433.9; *P* < .001) (Table 1). A respiratory CCC was present in 21.6% of readmitted patients (809 of 3748 children) compared with 11.0% (1078 of 9757 children) of those not readmitted (Pearson χ^2^_2_ = 531.9; *P* < .001). Discharge home was the most frequent disposition in both readmitted and not-readmitted children; however, discharge to a nonacute care facility occurred in 39.3% (1472 of 3748 children) of readmitted children compared with 21.5% (2101 of 9757 children) of those not readmitted.

**Table 1.** Index Hospital Characteristics for Children with Algorithm-Identified Acute Respiratory Distress Syndrome by 1-Year Readmission

Characteristic	Patients, No. (%)			Univariable Cox HR for readmission (95% CI)	*P* value
	Total (N = 13 505)	Not readmitted (n = 9757)	Readmitted (n = 3748)		
Length of stay, median (IQR), d	12 (4-36)	10 (4-28)	19 (7-55)	NA	NA
Length of stay (categorical), d					
≤1	1333 (9.9)	1041 (10.7)	292 (7.8)	1 [Reference]	NA
2-3	1647 (12.2)	1324 (13.6)	323 (8.6)	0.91 (0.77-1.08)	.28
4-7	2184 (16.2)	1771 (18.2)	413 (11.0)	0.86 (0.73-1.02)	.08
8-13	2127 (15.7)	1603 (16.4)	524 (14.0)	1.17 (1.00-1.38)	.049
≥14	6214 (46.0)	4018 (41.2)	2196 (58.6)	1.82 (1.58-2.09)	<.001
Sex at birth					
Male	7869 (58.3)	5723 (58.7)	2146 (57.3)	1 [Reference]	NA
Female	5636 (41.7)	4034 (41.3)	1602 (42.7)	1.03 (0.96-1.10)	.44
Age, y					
<1	5305 (39.3)	3772 (38.7)	1533 (40.9)	Reference	NA
1-4	2577 (19.1)	1754 (18.0)	823 (22.0)	1.11 (1.02-1.22)	.02
5-12	2154 (15.9)	1617 (16.6)	537 (14.3)	0.82 (0.74-0.91)	<.001
13-18	3469 (25.7)	2614 (26.8)	855 (22.8)	0.86 (0.79-0.94)	.001
Complex chronic condition category					
None	4956 (36.7)	4103 (42.1)	853 (22.8)	1 [Reference]	NA
Nonrespiratory	6662 (49.3)	4576 (46.9)	2086 (55.7)	2.04 (1.88-2.22)	<.001
Respiratory	1887 (14.0)	1078 (11.0)	809 (21.6)	3.01 (2.73-3.33)	<.001
Public insurance (Medicaid)	8455 (62.6)	5974 (61.2)	2481 (66.2)	1.07 (1.00-1.15)	.06
Tracheostomy during index hospitalization	355 (2.6)	166 (1.7)	189 (5.0)	2.46 (2.11-2.87)	<.001
Discharge disposition					
Home	8968 (66.4)	6944 (71.2)	2024 (54.0)	1 [Reference]	NA
Inpatient rehabilitation	554 (4.1)	373 (3.8)	181 (4.8)	1.64 (1.41-1.92)	<.001
Nonacute care facility	3573 (26.5)	2101 (21.5)	1472 (39.3)	2.22 (2.07-2.39)	<.001
Hospice	19 (0.1)	10 (0.1)	9 (0.2)	3.04 (1.45-6.38)	.003

Abbreviations: HR, hazard ratio; NA, not applicable.

**Figure 2.**
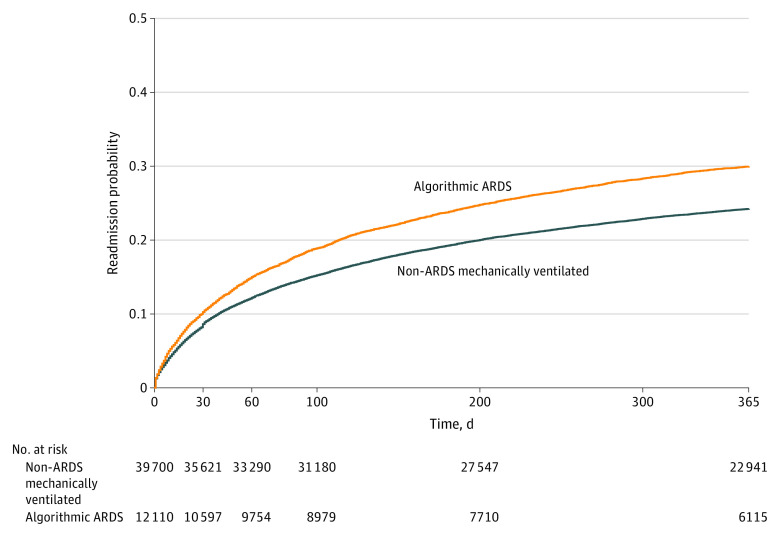
Kaplan-Meier Survival Curve Comparing Readmission for Children Receiving Mechanical Ventilation With Those With Algorithm-Identified Acute Respiratory Distress Syndrome (ARDS)

The probability of readmission for all recipients of MV was 9.0% (95% CI, 8.7%-9.2%) at 30 days and increased to 25.6% (95% CI, 25.2%-25.9%) at 1 year. For children meeting algorithmic ARDS criteria, the probability of readmission was significantly higher, with a 30-day readmission of 10.2% (95% CI, 9.7%-10.8%; log-rank *P* < .001), increasing to 30.0% (95% CI, 29.0%-30.8%) at 1 year (Figure 2). One-third of ARDS readmissions (or 10% of the total ARDS cohort population) occurred within the first 30 days after index discharge (95% CI, 27-32 days), and one-half of ARDS readmissions (or 15% of the total ARDS cohort population) were within 61 days of discharge (95% CI, 56-67 days).

For 3748 children readmitted after the index ARDS hospitalization, receipt of respiratory support during readmission was common (2334 children [62.3%]); 1280 readmitted children (34.2%) again met the criteria for algorithm-identified ARDS. A diagnosis of pneumonia was present in 34.5% of readmissions (1304 readmissions), bronchiolitis in 2.1% (79 readmissions), and severe sepsis or shock in 8.0% (298 readmissions). Readmission diagnoses differed by age categorization for both pneumonia (Pearson χ^2^_3_ = 45.3; *P* < .001) and bronchiolitis (Pearson χ^2^_3_ = 59.8; *P* < .001).

**Figure 3.**
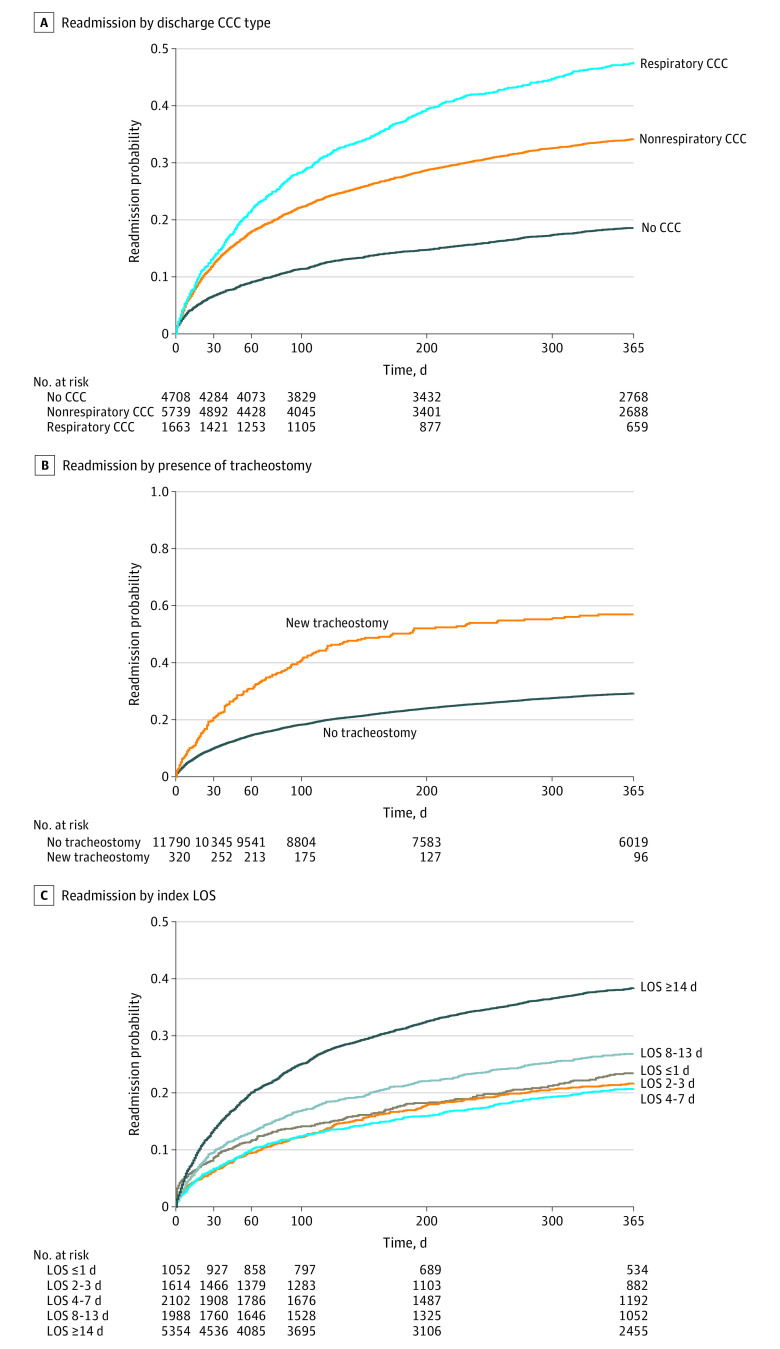
Kaplan-Meier Survival Curves of Unadjusted Readmission by Primary Exposures of Interest Graphs show probability of readmission after algorithmic-identified acute respiratory distress syndrome according to type of complex chronic condition (CCC) present at discharge (A), whether new tracheostomy was performed during hospitalization (B), and hospitalization length of stay (LOS) (C).

Univariable associations with readmission are presented in Table 1. After controlling for confounders, nonrespiratory and respiratory CCCs were associated with increased hazard of readmission at 30 days, 60 days, and 1 year (Table 2) (Figure 3A). Compared with nonrespiratory CCC (adjusted hazard ratio [aHR], 1.86; 95% CI, 1.71-2.03), respiratory CCC (aHR, 2.69; 95% CI, 2.42-2.98) was associated with significantly higher hazard of 1-year readmission (Pearson χ^2^_1_ = 66.6; *P* < .001). Receiving a tracheostomy during the index admission was associated with readmission at all time points (aHR, 1.98; 95% CI, 1.69-2.33) (Figure 3B). Admission LOS 14 days or longer was associated with significantly higher hazard of readmission at all time points (aHR, 1.87; 95% CI, 1.62-2.16). LOS 8 to 13 days was associated with increased hazard of readmission at 1 year (HR, 1.26; 95% CI, 1.07-1.48), but not at 30 or 60 days. At 1 year, the hazard of readmission for children with LOS 8 to 13 days was significantly higher than for a 4- to 7-day admission (Pearson χ^2^_1_ = 20.4; *P* < .001). LOS 7 days or less was not associated with readmission (Figure 3C).

**Table 2.** Multivariable Hazard for 3 Exposures of Interest at 30 and 60 Days and 1-Year Readmission

Variable	Adjusted HR (95% CI)		
	Readmission at 30 d	Readmission at 60 d	Readmission at 1 y
Complex chronic condition category			
None	1 [Reference]	1 [Reference]	1 [Reference]
Nonrespiratory	1.46 (1.30-1.65)	1.56 (1.42-1.73)	1.86 (1.71-2.03)
Respiratory	1.53 (1.30-1.79)	1.76 (1.55-1.99)	2.69 (2.42-2.98)^a^
Tracheostomy during index hospitalization	1.47 (1.15-1.88)	1.54 (1.26-1.89)	1.98 (1.69-2.33)
Length of index hospitalization, d			
0-1	1 [Reference]	1 [Reference]	1 [Reference]
2-3	0.78 (0.61-1.00)	0.83 (0.68-1.01)	0.97 (0.81-1.15)
4-7	0.84 (0.67-1.06)	0.90 (0.75-1.09)	0.93 (0.78-1.09)
8-13	1.05 (0.84-1.31)^b^	1.03 (0.86-1.24)	1.26 (1.07-1.48)^b^
≥14	1.38 (1.13-1.68)^c^	1.42 (1.21-1.67)^c^	1.87 (1.62-2.16)^c^

Abbreviation: HR, hazard ratio.

^a^ Denotes a significant difference between nonrespiratory and respiratory conditions.

^b^ Denotes a significant difference between 4 to 7 days and 8 to 13 days.

^c^ Denotes a significant difference between 8 to 13 days and 14 days or longer.

Excluding children with immunocompromising conditions, respiratory and nonrespiratory CCCs, new tracheostomy, and LOS greater than 7 days all continued to be associated with 1-year readmission (eTable 2 in Supplement 1). Supplementary traditional risk factor multivariable Cox and exponential distribution models with all available covariates were constructed (eTable 3 in Supplement 1). For 4568 children with no CCC, index LOS 14 days or longer (aHR, 1.92; 95% CI, 1.49-2.47) (eTable 4 in Supplement 1) was associated with readmission. After excluding children with a diagnosis of asthma or status asthmaticus, both categorizations of CCC, new tracheostomy, and LOS 8 to 13 days (aHR, 1.27; 95% CI, 1.08-1.50) and 14 days or longer (aHR, 1.89; 95% CI, 1.63-2.19) were associated with increased hazard of readmission (eTable 5 in Supplement 1).

In adjusted analyses, children with nonrespiratory CCCs had 23.7 fewer DAOH (95% CI, 18.9-28.4 DAOH), and those with respiratory CCCs had 25.9 fewer DAOH (95% CI, 20.2-31.5 DAOH) after index discharge (eTables 6 and 7 in Supplement 1). In adjusted analyses, both new tracheostomy and LOS 14 days or longer were associated with reduced DAOH (eTable 7 in Supplement 1).

## Discussion

This cohort study found that readmission in the first year after ARDS was common in children, with a 1-year probability of readmission of 30.0%. Key factors associated with 1-year readmission include being identified as having a new or existing CCC at the time of index discharge, new tracheostomy during index admission, and LOS greater than 7 days. Determining patient-level index hospitalization factors associated with readmission that are identifiable at discharge offers the opportunity for targeting future readmission mitigation strategies to those most at risk, with important implications for the health care system.

We found that children who received MV for any reason and of any duration were readmitted more frequently (25.6%) by 1 year than both the general PICU population (11.0%)^10^ and the general pediatric population (7.0%).^10,25^ Children with algorithmic-determined ARDS were readmitted even more frequently than the general MV population, but less often than children with severe sepsis.^11^ Our findings demonstrate that children who survive ARDS may have lasting impacts on their health in the form of the burdens of readmission and its sequalae.

We found that nearly one-half of readmissions occurred in the first 2 months following index discharge, suggesting that the 2-month period immediately after discharge is an important window for early intervention. In some health care systems, additional support to all PICU survivors may be feasible, whereas in others, targeted approaches to higher risk populations may be more practical. With the increasing focus on post–intensive care syndrome,^26,27^ all children who received MV, but especially those with ARDS, may be ideal for future postdischarge follow-up clinics or subspecialty referrals, with appointments scheduled within this high-risk period soon after discharge.

More specifically, our findings also highlight that among ARDS survivors, children who have a CCC, new tracheostomies, and a LOS 14 days or longer are particularly at risk of readmission. We also found that children with a respiratory CCC were more likely to be readmitted by 1 year compared with children with a nonrespiratory CCC. It is unknown whether this finding is unique to children with index hospitalization for pediatric ARDS or whether children with respiratory CCC are an especially high-risk group regardless of their hospitalization principal problem. Future work for children with CCCs may benefit from examining individual domains of CCCs in addition to the overall presence of any CCC. Similarly, patients requiring tracheostomy during their ARDS course also deserve special consideration, regardless of whether they have additional CCCs. These children are at high risk of postdischarge complications and pose a high financial burden on families and the health care system, and any socioeconomic disparities in their families may further exacerbate disparate outcomes in these children.^28,29^ Finally, our data further support a dose-response relationship between LOS and hazard of readmission, at both 30 days and 1 year.

Our findings support focusing on known mitigators of readmission, such as home health care and early follow-up, and trials of novel readmission mitigation strategies on children with respiratory CCCs, new tracheostomies, and longer LOS. Efforts to reduce readmissions could have major implications on the pediatric health care system. Improving the postdischarge safety net may include further access to home health care, which has been shown to be less available for children with chronic medical issues than their adult counterparts.^30^ In addition, attempted contact to discuss issues arising after discharge has been shown to reduce 30-day readmission in geriatric patients,^31^ and earlier follow-up appointments have been associated with decreased readmission for children with chronic conditions.^32^ The establishment of early postdischarge contact, either via telephone call or text message or with a scheduled follow-up visit, are feasible interventions that should be implemented and evaluated.

### Limitations

This study has limitations that should be mentioned. The algorithm to identify ARDS was initially constructed with *ICD-10* codes^16^ and was designed to have high specificity with good sensitivity. We used conversion tables to account for the years with *ICD-9* coding in our data set, but exposure misclassification of ARDS may have occurred. Not all children had pre–index hospitalization records that could be used to determine the presence of a CCC. Discharge determination of CCC may be affected by misclassification bias owing to inadequate *ICD-9* or *ICD-10* coding or diagnoses that resolved during hospitalization and were not present at discharge. Future studies with the granularity of data to disentangle severity of illness measures, the presence of a CCC before admission, and new CCC present at discharge would help to further refine at-risk groupings. The benefits of single-center or limited-center studies must be balanced with the limitations of not including different hospital readmissions, which was previously reported by Khan et al^15^ to be as high as 13.9% of pediatric readmissions. We limited our study to children with 30 days of coverage before the index hospitalization to avoid including patients’ enrolling in a health care plan midhospitalization and inaccurate admission dating. Our data set did not capture patients without insurance; therefore, this at-risk population was not included in analyses. Our study found that ARDS readmission probability was between that of the general PICU population^10^ and children with severe sepsis.^11^ Although ARDS is defined as a pulmonary disease, the sequalae of ARDS may be related to the complex interplay of ARDS severity and its multisystem effects. Intubation and MV require sedation, and sometimes neuromuscular blockade. Neurologic comorbidity, neuromuscular comorbidity, and potentially a predilection for future infections may be a consequence of ARDS. Unfortunately, beyond *ICD-10* diagnosis and procedure codes, granular determination of severity of illness or organ failures was not available. Outpatient mortality is a competing risk of our primary outcome of readmission, but we did not have access to outpatient mortality records. Reassuringly, pediatric outpatient deaths are uncommon, even in children with CCC, and we have previously reported the 1-year mortality after ARDS to be low.^33,34^ We performed sensitivity analyses for readmission at 30 and 60 days and used an exponential distribution to address concerns of nonproportionality. In addition, we used insurance-based administrative data sets to mitigate selection bias in previous studies relying on readmission to the same hospital but potentially missed readmissions in censored patients with change of insurance coverage before 1 year. Our primary outcome examined factors associated with first readmission but did not account for subsequent readmissions or time spent readmitted. To address this concern, we evaluated DAOH after discharge and found that CCCs, new tracheostomy, and LOS 14 days or longer were associated with significant reductions in DAOH in the first year after discharge.

Socioeconomic and racial disparities have been shown to be important factors in health care utilization and readmission in children with asthma.^35,36^ Our access to socioeconomic data was limited to public vs private insurance, and racial data were not available. We did not find an association between hazard of readmission in either univariable or multivariable analyses for children on Medicaid. We recognize that this does not exclude that a more nuanced evaluation may determine that socioeconomic factors are important considerations in readmission after ARDS.

## Conclusions

In children with ARDS who survive to hospital discharge, the probability of readmission is 30.0% at 1 year, higher than previously reported for the general PICU population. Important factors for readmission include chronic medical conditions, including immunocompromising diseases, new tracheostomy placement, and an index hospitalization of 14 days or longer. Targeted interventions in both chronically ill and previously healthy children may best focus on children who are hospitalized for longer than 2 weeks. One-half of readmissions occurred within 61 days of hospital discharge, suggesting that interventions during the early postdischarge period might have an impact on outcomes trajectories. Future studies are needed to evaluate whether such interventions as postdischarge telephonic contact, follow-up clinics, and home health care may reduce the readmission burden facing pediatric ARDS survivors.

## Notes

Supplement 1. **eTable 1.** International Classification of Disease Codes and Algorithms **eTable 2.** Multivariable Hazard for 3 Exposures of Interest for One-Year Readmission Excluding Children With an Immunocompromising Condition **eTable 3.** Risk Factor Multivariable Cox Proportional Hazard and Exponential Distribution Model for One-Year Readmission **eTable 4.** Multivariable Hazard of Length-of-Stay on One-Year Readmission for Children Without a Complex Chronic Condition **eTable 5.** Multivariable Hazard for 3 Exposures of Interest for One-Year Readmission Excluding Children With Asthma **eTable 6.** Days Alive and Out of Hospital After Index Discharge **eTable 7.** Differences in Predicted Days Alive and Out of Hospital After Discharge for Children With Algorithmic ARDS **eFigure 1.** Directed Acyclic Graph **eFigure 2.** Violin Plot of Length of Stay for Children With Algorithm Identified ARDS by One-Year Readmission

Supplement 2.
Data Sharing Statement
