# Supplementary material for: Readmission Rates After Acute Respiratory Distress Syndrome in Children
Source: JAMA Netw Open. 2023 Sep 8;6(9):e2330774. doi: 10.1001/jamanetworkopen.2023.30774 (PMC10492185; doi:10.1001/jamanetworkopen.2023.30774)
Supplement: Supplement 1. — eTable 1. International Classification of Disease Codes and Algorithms eTable 2. Multivariable Hazard for 3 Exposures of Interest for One-Year Readmission Excluding Children With an Immunocompromising Condition eTable 3. Risk Factor Multivariable Cox Proportional Hazard and Exponential Distribution Model for One-Year Readmission eTable 4. Multivariable Hazard of Length-of-Stay on One-Year Readmission for Children Without a Complex Chronic Condition eTable 5. Multivariable Hazard for 3 Exposures of Interest for One-Year Readmission Excluding Children With Asthma eTable 6. Days Alive and Out of Hospital After Index Discharge eTable 7. Differences in Predicted Days Alive and Out of Hospital After Discharge for Children With Algorithmic ARDS eFigure 1. Directed Acyclic Graph eFigure 2. Violin Plot of Length of Stay for Children With Algorithm Identified ARDS by One-Year Readmission [file jamanetwopen-e2330774-s001.pdf]

Supplemental Online Content

Keim G, Hsu JY, Pinto NP, et al. Readmission rates after acute respiratory distress syndrome in children. *JAMA Netw Open*. 2023;6(8):e2330774. doi:10.1001/jamanetworkopen.2023.30774

eTable 1. International Classification of Disease Codes and Algorithms
eTable 2. Multivariable Hazard for 3 Exposures of Interest for One-Year Readmission Excluding Children With an Immunocompromising Condition
eTable 3. Risk Factor Multivariable Cox Proportional Hazard and Exponential Distribution Model for One-Year Readmission
eTable 4. Multivariable Hazard of Length-of-Stay on One-Year Readmission for Children Without a Complex Chronic Condition
eTable 5. Multivariable Hazard for 3 Exposures of Interest for One-Year Readmission Excluding Children With Asthma
eTable 6. Days Alive and Out of Hospital After Index Discharge
eTable 7. Differences in Predicted Days Alive and Out of Hospital After Discharge for Children With Algorithmic ARDS
eFigure 1. Directed Acyclic Graph
eFigure 2. Violin Plot of Length of Stay for Children With Algorithm Identified ARDS by One-Year Readmission

This supplemental material has been provided by the authors to give readers additional information about their work.

© 2023 Keim G et al. *JAMA Network Open.*

eTable 1: International Classification of Disease Codes and Algorithms

Diagnosis	ICD-10 Codes	ICD-9 Codes
Acute Respiratory Distress Syndrome	J80*, J81*	518.82
Asthma	J45*	493*
Pneumonia	J09*, J10*, J11*, J12*, J13*, J14*, J15*, J16*, J17*, J18*	480*, 481*, 482*, 483*, 484*, 485*, 486*, 487*, 488*
Bronchiolitis	J20*, J21*, J22*, J40	466*
Pneumonitis	J68*, J69*	506*
Other respiratory failure	J96*, R06.03	518.51, 518.84
Mechanical Ventilation (Diagnosis)	Z99.11	-
Sepsis/ Shock	A41*, R57*, R65.2*	995.9*, 785.52, 785.50
Seizure and Status Epilepticus	G40.901, R56.9*, G40.001, G40.101, G40.111	345.3*
Procedure		
Invasive Mechanical Ventilation <24 Hours	5A19035	967.1
Invasive Mechanical Ventilation ≥24 Hours	5A19045, 5A19055	967.2
Endotracheal Intubation	0BH17EZ	960.4
Algorithm	Code Composition	
Algorithm Identified ARDS	(5A19045 or 5A19055) + (Any Diagnosis Code for Acute Respiratory Distress Syndrome, Asthma, Pneumonia, Bronchiolitis, Pneumonitis, Other respiratory failure, Sepsis/ Shock) + (NONE of G40.901 or R56.9*or G40.001 or G40.101 or G40.111)	(967.2) + (Any Diagnosis Code for Acute Respiratory Distress Syndrome, Asthma, Pneumonia, Bronchiolitis, Pneumonitis, Other respiratory failure, Sepsis/ Shock) + (NONE of 345.3*)

*Denotes that any suffix code was allowed

© 2023 Keim G et al. *JAMA Network Open.*

eTable 2: Multivariable Hazard for 3 Exposures of Interest for One-Year Readmission Excluding Children With an Immunocompromising Condition

	One-Year
Variable	Adjusted Hazard Ratio (95%CI)
Complex Chronic Condition Category	
None	Reference
Non-Respiratory	1.86 (1.70 – 2.02)
Respiratory	2.68 (2.42 – 2.98)*
Tracheostomy During Index Hospitalization	1.97 (1.67 – 2.31)
Length of Index Hospitalization	
0-1 Days	Reference
2-3 Days	0.97 (0.82 – 1.16)
4-7 Days	0.92 (0.78 – 1.09)
8-13 Days	1.26 (1.07 – 1.48) δ
≥ 14 Days	1.86 (1.60 – 2.15)^

* Significant difference between Non-Respiratory and Respiratory
δ Significant difference between 4-7 days and 8-13 days
^ Significant difference between 8-13 days and ≥14 days

© 2023 Keim G et al. *JAMA Network Open.*

eTable 3: Risk Factor Multivariable Cox Proportional Hazard and Exponential Distribution Model for One-Year Readmission

	Cox Proportional Hazard	Exponential Distribution Hazard
Variable	Adjusted Hazard Ratio (95%CI)	Adjusted Hazard Ratio (95%CI)
Age (Years)		
<1	Reference	Reference
1-4	1.53 (1.39 – 1.69)	1.59 (1.45 – 1.75)
5-12	1.15 (1.03 – 1.29)	1.14 (1.02 – 1.28)
13-17	1.25 (1.13 – 1.39)	1.28 (1.16 – 1.42)
Length of Index Hospitalization		
0-1 Days	Reference	Reference
2-3 Days	0.93 (0.78 – 1.11)	0.95 (0.79 – 1.13)
4-7 Days	0.89 (0.75 – 1.05)	0.88 (0.77 – 1.04)
8-13 Days	1.20 (1.02 – 1.41)	1.22 (1.04 – 1.44)
≥ 14 Days	1.62 (1.40 – 1.88)	1.69 (1.46 – 1.96)
Public Insurance	0.95 (0.88 – 1.02)	0.89 (0.83 – 0.96)
Complex Chronic Condition		
Non-Respiratory	1.70 (1.56 – 1.86)	1.78 (1.63 – 1.94)
Respiratory	2.48 (2.23 – 2.76)	2.71 (2.44 – 3.01)
Immunodeficiency	2.38 (1.80 – 3.16)	2.75 (2.08 – 3.64)
Tracheostomy During Index Hospitalization	1.17 (0.99 – 1.39)	1.25 (1.06 – 1.48)
Discharge Disposition		
Home	Reference	Reference
Inpatient Rehabilitation	1.34 (1.13 – 1.58)	1.38 (1.17 – 1.75)
Transfer to Non-Acute Care Facility	1.90 (1.77 – 2.05)	2.05 (1.91 – 2.21)
Hospice	2.18 (1.04 – 4.59)	2.83 (1.34 – 5.95)

© 2023 Keim G et al. *JAMA Network Open.*

eTable 4: Multivariable Hazard of Length-of-Stay on One-Year Readmission for Children Without a Complex Chronic Condition

	One-Year
Variable	Adjusted Hazard Ratio (95%CI)
Length of Index Hospitalization	
0-1 Days	Reference
2-3 Days	0.93 (0.69 – 1.24)
4-7 Days	0.85 (0.64 – 1.13)
8-13 Days	1.25 (0.95 – 1.65) $^{\delta}$
≥ 14 Days	1.92 (1.49 – 2.47)^

δ Significant difference between 4-7 days and 8-13 days

^ Significant difference between 8-13 days and ≥14 days

© 2023 Keim G et al. *JAMA Network Open.*

eTable 5: Multivariable Hazard for 3 Exposures of Interest for One-Year Readmission Excluding Children With Asthma

	One-Year
Variable	Adjusted Hazard Ratio (95%CI)
Complex Chronic Condition Category	
None	Reference
Non-Respiratory	1.87 (1.72 – 2.05)
Respiratory	2.73 (2.46 – 3.03)
Tracheostomy During Index Hospitalization	1.98 (1.68 – 2.32)
Length of Index Hospitalization	
0-1 Days	Reference
2-3 Days	0.99 (0.83 – 1.18)
4-7 Days	0.94 (0.79 – 1.11)
8-13 Days	1.27 (1.08 – 1.50)
≥ 14 Days	1.89 (1.63 – 2.19)

© 2023 Keim G et al. *JAMA Network Open.*

eTable 6: Days Alive and Out of Hospital After Index Discharge

	Days (Interquartile Range)	p- value
Complex Chronic Condition Category		p= 0.0001
None	342 (317 – 354)	
Non-Respiratory	310 (256 – 342)	
Respiratory	326 (258 – 351)	
No New Tracheostomy	328 (272 – 349)	P<0.0001
New Tracheostomy	277 (212 – 320)	
Length of Index Hospitalization		P=0.0001
0-1 Days	354 (334 – 361)	
2-3 Days	355 (341 – 360)	
4-7 Days	352 (339 – 356)	
8-13 Days	347 (334 – 351)	
≥ 14 Days	286 (239 – 326)	

© 2023 Keim G et al. *JAMA Network Open.*

eTable 7: Differences in Predicted Days Alive and Out of Hospital After Discharge for Children With Algorithmic ARDS

Variable	Unadjusted Decrease in Predicted Days Out of Hospital (95% CI)	Adjusted Decrease in Predicted Days Out of Hospital (95% CI)
Complex Chronic Condition Category		
None	Reference	Reference
Non-Respiratory	39.5 (34.4 – 44.6)	23.7 (18.99 – 28.4)
Respiratory	31.0 (24.8 – 37.1)	25.9 (20.2 – 31.5)
Tracheostomy During Index Hospitalization	42.3 (32.7 – 51.8)	50.6 (42.1 – 59.2)
Length of Index Hospitalization		
0-1 Days	Reference	Reference
2-3 Days	3.5 (5.4 – 12.5*)	12.9 (4.4 – 21.3)
4-7 Days	3.6 (4.9 – 12.1*)	16.1 (8.0 – 24.1)
8-13 Days	2.72 (10.8 – 5.39)	17.7 (10.1 – 25.4)
≥ 14 Days	66.7 (59.7 – 73.6)	64.6 (58.0 – 71.2)

* Represents increase in Days Alive and Out of Hospital

© 2023 Keim G et al. *JAMA Network Open.*

eFigure 1: Directed Acyclic Graph

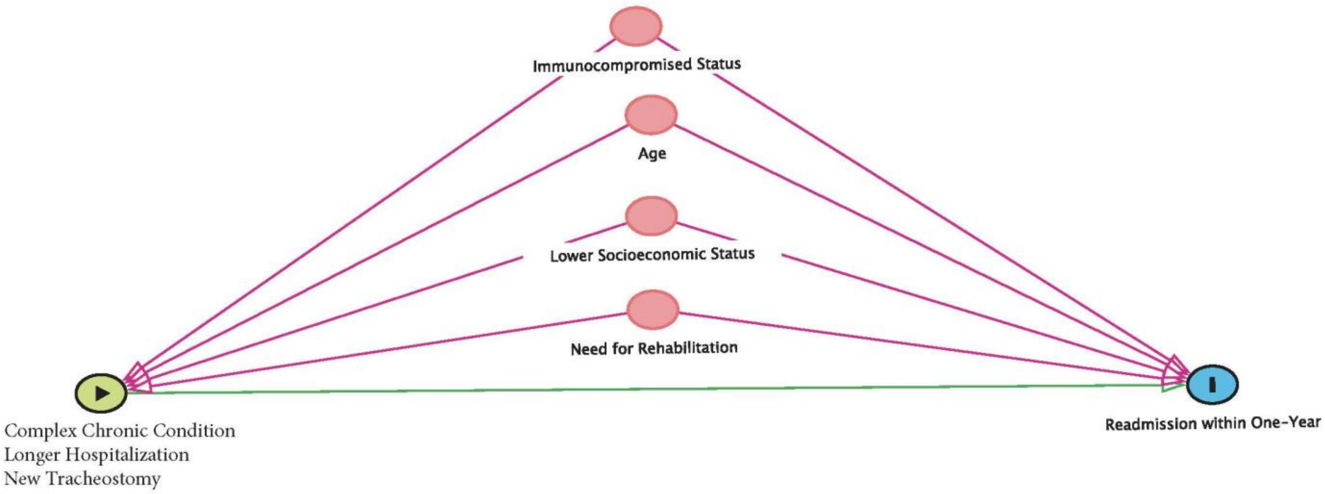

© 2023 Keim G et al. *JAMA Network Open.*

eFigure 2: Violin Plot of Length of Stay for Children With Algorithm Identified ARDS by One-Year Readmission

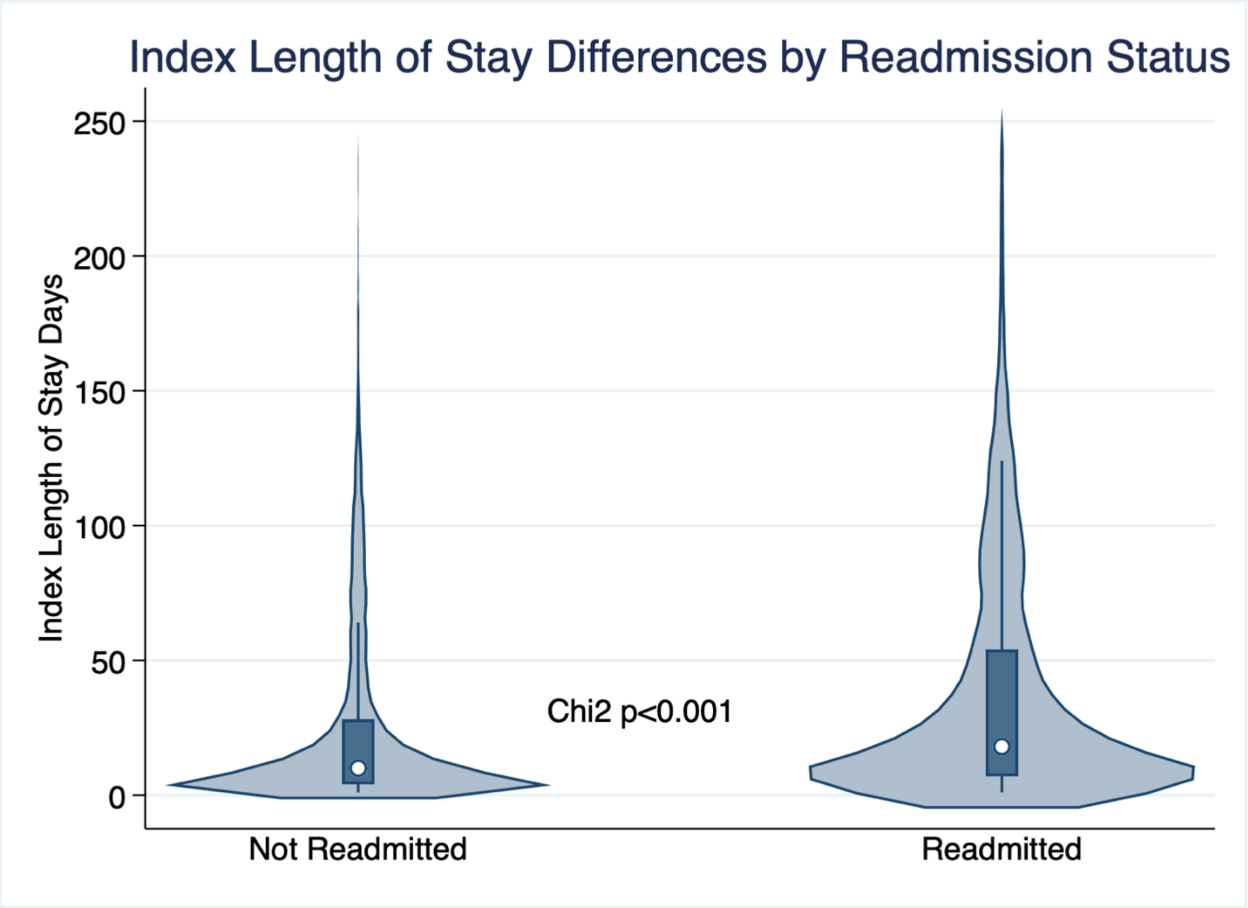

© 2023 Keim G et al. *JAMA Network Open.*
