# Supplementary material for: Readmission Rates After Acute Respiratory Distress Syndrome in Children
Source: JAMA Netw Open. 2023 Sep 8;6(9):e2330774. doi: 10.1001/jamanetworkopen.2023.30774 (PMC10492185; doi:10.1001/jamanetworkopen.2023.30774)
Supplement: Supplement 2. — Data Sharing Statement [file jamanetwopen-e2330774-s002.pdf]

Data Sharing Statement

Keim. Readmission Rates After Acute Respiratory Distress Syndrome in Children. *JAMA Netw Open*. Published August 31, 2023. doi:10.1001/jamanetworkopen.2023.30774

Data
Data available: Yes
Data types: Deidentified participant data, Data dictionary
How to access data: Requests for data should be sent to keimg@chop.edu
When available: With publication

Supporting Documents
Document types: Statistical/analytic code
How to access documents: Requests for data should be sent to keimg@chop.edu
When available: With publication

Additional Information
Who can access the data: Researchers with an approved proposal will have data made available to them upon request
Types of analyses: Data can be made available for any purpose
Mechanisms of data availability: Data will be made available after approval of proposal with investigator support available if appropriate
